# Plant species distributions along environmental gradients: do belowground interactions with fungi matter?

**DOI:** 10.3389/fpls.2013.00500

**Published:** 2013-12-10

**Authors:** Loïc Pellissier, Eric Pinto-Figueroa, Hélène Niculita-Hirzel, Mari Moora, Lucas Villard, Jérome Goudet, Nicolas Guex, Marco Pagni, Ioannis Xenarios, Ian Sanders, Antoine Guisan

**Affiliations:** ^1^Department of Ecology and Evolution, University of LausanneLausanne, Switzerland; ^2^Institute for Work and Health, University of Lausanne and GenevaLausanne, Switzerland; ^3^Department of Botany, Institute of Ecology and Earth Sciences, University of TartuTartu, Estonia; ^4^VitalIT, Swiss Institute of BioinformaticsLausanne, Switzerland; ^5^Institute of Earth Sciences, University of LausanneLausanne, Switzerland

**Keywords:** fungal communities, plant assemblage, elevation, 454 pyrosequencing, species distribution models

## Abstract

The distribution of plants along environmental gradients is constrained by abiotic and biotic factors. Cumulative evidence attests of the impact of biotic factors on plant distributions, but only few studies discuss the role of belowground communities. Soil fungi, in particular, are thought to play an important role in how plant species assemble locally into communities. We first review existing evidence, and then test the effect of the number of soil fungal operational taxonomic units (OTUs) on plant species distributions using a recently collected dataset of plant and metagenomic information on soil fungi in the Western Swiss Alps. Using species distribution models (SDMs), we investigated whether the distribution of individual plant species is correlated to the number of OTUs of two important soil fungal classes known to interact with plants: the Glomeromycetes, that are obligatory symbionts of plants, and the Agaricomycetes, that may be facultative plant symbionts, pathogens, or wood decayers. We show that including the fungal richness information in the models of plant species distributions improves predictive accuracy. Number of fungal OTUs is especially correlated to the distribution of high elevation plant species. We suggest that high elevation soil show greater variation in fungal assemblages that may in turn impact plant turnover among communities. We finally discuss how to move beyond correlative analyses, through the design of field experiments manipulating plant and fungal communities along environmental gradients.

## Effects of mycorrhizal fungi on plant species distributions: a review of evidence

Plant species are not randomly distributed in the landscape along environmental gradients (Pottier et al., 2013), but display species-specific tolerances shaping non-random assemblages (Kikvidze et al., 2005). Among abiotic drivers, climate and soil properties are recognized to strongly constrain the distribution of species (e.g., Dubuis et al., 2013). Species are not an independent entity in an ecosystem, but interact ecologically positively (e.g., facilitation, mutualism) and negatively (e.g., competitive exclusion; Pellissier et al., 2012). The search for principles explaining the local assembly of communities should thus integrate biotic processes (Guisan and Rahbek, 2011; Weiher et al., 2011). In the same way as abiotic variables determine species distributions, biotic interactions constrain individual species ranges and, thus, the spatial variation in species assemblages (Wisz et al., 2013). Fungi display mutualistic and antagonistic interactions with plants and influence plant growth (Smith and Read, 2008). As fungal distribution and community composition are also patterned along abiotic gradients (Opik et al., 2010; Tedersoo et al., 2012), we expect that in addition to climatic and soil abiotic factors, shifts in fungal assemblages structure along ecological gradients should further constrain plant species distributions.

The majority of plants establish mycorrhizal symbioses with fungi. Some of these symbiotic fungi, the arbuscular mycorrhizal fungi (AMF), are obligate biotrophs that require the host plant to complete their life cycle (Smith and Read, 2008). Others, such as ectomycorrhizal fungi (ECM), are less dependent on the host plant. In these associations, the fungus supplies the plant with inorganic nutrients, such as nitrate and phosphate, and the plant provides the fungal partners with photosynthates (Li et al., 2006). Plant-fungal symbioses influence different key aspects of plant life such as clonal reproduction (Streitwolf-Engel et al., 2001), growth and reproductive success (Sanders, 2010). In addition, symbioses with AMF significantly increase the tolerance of the host plant to abiotic stress such as drought and salinity, and to biotic stress including above- and below-ground pathogens and parasites (Whipps, 2004; Jung et al., 2012). Also, AMF have been shown to have a positive effect on the association of the plant with nitrogen-fixing bacteria or phosphate-solubilizing bacteria (Jung et al., 2012). The presence of ECM fungal species may affect plant fitness by regulating the nitrate content of the soil. Indeed, these fungal species have the capacity to immobilize nitrates within their cytoplasm and deliver it to plant partners in a manner independent of soil available nitrate levels. Given how mycorrhizae affect individual plants, mycorrhizal fungal community structure is expected to modulate both the plant's competitive abilities and tolerance to abiotic conditions, and therefore the composition of plant communities (van der Heijden et al., 1998; van der Heijden and Sanders, 2002).

Mycorrhizal effects on plant communities range from a shift in species relative abundance to modification of plant community composition and diversity (Grime et al., 1987; Hartnett and Wilson, 1999; van der Heijden, 2003). The mycorrhizal symbiosis can also alter plant community structure by shifting the competitive balance between plant species within communities and by providing relative advantages to plant species that may otherwise be inferior competitors (Moora and Zobel, 2010). In particular, the effect of arbuscular mycorrhizae on plants can be especially pronounced at the seedling establishment stage (Francis and Read, 1995), which is crucial in shaping local species composition and diversity of plant communities (Dickson and Foster, 2008; Koorem et al., 2012). For instance, Koorem et al. (2012) suggest that mycorrhization of *Oxalis acetosella* and *Prunella vulgaris* increases their ability to establish in low soil fertility conditions. Other attempts to evaluate the interactions between fungal and plant communities were performed using fungicide treatments in experiments and documented shifts in community composition following application (Hartnett and Wilson, 1999; Liu et al., 2012). Although the potential role of mycorrhizae in structuring plant communities has been discussed for decades (Zobel et al., 1997), evidence of this role extending beyond the scale of the community remains scarce (Klironomos et al., 2011).

Soil fungi, and particularly mycorrhizal fungi, are expected to play a key role in determining large-scale distribution of plant species (Fitter et al., 2005; Rosendahl, 2008; van der Heijden et al., 2008). The failure of many ectomycorrhizal plants to establish in the absence of ECM suggests that the effect of ECM on plant distribution and assemblages is likely to extend at large spatial scale (Klironomos et al., 2011). Recent studies show that the richness of ECM fungi is related to temperature and precipitation at the global scale, peaking in temperate and boreal forest biomes (Tedersoo et al., 2012). This may constrain the global distribution of plants associated with ECM fungi. Nevertheless, while the distribution of plant species along environmental gradients is relatively well described, the biogeography of microorganisms, like soil fungi, is in its infancy. Although some advances have been made (e.g., Moora et al., 2004, 2011; Fitter et al., 2005; Treseder and Cross, 2006; Chaudhary et al., 2008; Kivlin et al., 2011; Tedersoo et al., 2012; Turrini and Giovannetti, 2012; Yang et al., 2012; Opik et al., 2013), due to the cryptic lifestyle of soil fungi, knowledge of the global patterns of species distributions remains scarce. Our understanding of the co-variation of plants and their associated fungal communities at a landscape scale is still limited.

Invasive plant species may serve as a good example to understand the relationship between plants and patterns of the distribution of their fungal symbionts. A well-known example concerning the importance of mycorrhizal fungi comes from the *Pinus* genus following introduction to the southern hemisphere. While this genus is currently considered among the most invasive group of trees (Richardson and Rejmánek, 2004), initial plantings of *Pinus* sp. in the southern hemisphere failed due to the lack of the right ECM fungal species (Pringle et al., 2009; Dickie et al., 2010). The global spread of commercial ECM fungal inoculum has largely overcome this limitation (Vellinga et al., 2009), suggesting that the absence of coevolved mutualists was an important limitation to pine establishment before the introduction of ECM fungi. Arbuscular mycorrhizal fungi associate with the majority of plant species, are globally distributed and are generally believed to exhibit low host specificity (Smith and Read, 2008). As such, they may appear unlikely candidates to play a major role in plant invasions (Richardson et al., 2000). However, the global distribution of AMF taxa is challenged by the finding that in fact the majority of taxa highlighted by molecular analyses exhibit limited distribution (Opik et al., 2010). Consequently, plant species distributions at large scale could be constrained by the spatially restricted distribution of AMF taxa as shown for ECM fungi (Dickie et al., 2010).

One major caveat in our ability to understand the importance of fungal symbiosis in shaping plant distribution patterns is our limited understanding of the mycorrhizal status of most plant species (Reinhart et al., 2012). Several publications (Trappe, 1962; Harley and Harley, 1987; Wang and Qiu, 2006) report the presence of mycorrhizal fungi in the roots of a large number of plant species. However, a surprisingly small number of potential host plants are addressed. Hempel et al. (2013) show that for one of the best studied regions in the world, Central Europe, available information on the mycorrhizal status includes less than one third of the region's species (Wang and Qiu, 2006). Although simply documenting a plant species mycorrhizal status may not entirely reveal the mechanism concerning how a plant interacts with its symbiotic partner (Reinhart et al., 2012), it provides a basis for understanding landscape scale patterns in co-distribution (Wang and Qiu, 2006), Additionally, it has the potential of revealing ecological relationships between plants, their symbiotic fungi and the environment (Peat and Fitter, 1993; Hempel et al., 2013). Hempel et al. (2013) examined relationships between mycorrhizal status, habitat characteristics, life-history traits, and plant distribution patterns for 1752 members of the German flora. They showed that obligatorily mycorrhizal (OM) and non-mycorrhizal (NM) plant species tend to differ markedly from facultative mycorrhizal (FM) species in almost all analyzed criteria. Most interestingly, FM species show the widest geographic and ecological range, followed by OM and then NM species. This finding suggests that facultative interactions, including the ability to survive without a fungal mutualist when locally absent provides an advantage and ensures wider distributions. However, future studies about the co-occurrence of plants and fungi in natural environments will be required to understand the role of fungi in shaping plant distributions.

## Effects of soil fungi on plant species distributions: new insights from the Alps

Like plants, fungi also exhibit specific habitat requirements (Fitter et al., 2005). Therefore, at smaller scales, local drivers, such as soil characteristics, presumably play an important role. For example, mycorrhizal fungi are less abundant in moist, acidic and cold habitats (Peat and Fitter, 1993; Gavito et al., 2005) and more abundant under moderate nutrient stress (Smith and Read, 2008; Brundrett, 2009). Because assemblages of fungi potentially interacting with plants differ between environmental conditions, a shift in their distribution may be expected to affect both plant species distributions and community composition. However, this hypothesis, and the details of how fungi assemblage relates to plant species distributions, remained to be tested across many fungi and plant species and across various habitat types. In particular, Glomeromycetes AMFs are known to interact with most temperate grassland plant species (Harley and Harley, 1987; Wang and Qiu, 2006) and AMF assemblage is expected to influence plant tolerances to environmental factors as well as competitive abilities. Agaricomycetes, however, may function both as plant pathogens and as ECM fungi, and thus impact plant directly in either positive or negative ways. ECM fungi interact mostly with trees, but were also shown to form mutualisms with non-tree species such as alpine plants (Read and Haselwandter, 1981). Agaricomycetes may also impact plant communities indirectly as decayers, by affecting plant litter decay and nutrient availability (Zak et al., 2011).

One difficulty in detecting the effect of biotic interactions is that they usually co-vary with abiotic variables (Meier et al., 2010; Pellissier et al., 2010a,b). Fungal communities vary along the same abiotic gradients as plants. As for plants (Körner, 2003), variation in temperature (Bahram et al., 2012), precipitation (Hawkes et al., 2011) and snow cover (Zinger et al., 2009) may be associated with a shift in fungal community composition. Similarly, soil structure (Hartmann et al., 2008), acidity (Rousk et al., 2010), nitrogen content (Egerton-Warburton and Allen, 2000), and organic matter (Zinger et al., 2011) affect fungal community, composition just as they do in plant communities (Dubuis et al., 2013). Therefore, it is necessary to also account for abiotic variables when attempting to detect relationships between fungi and plant species distributions.

### Methods

**Figure 1:**
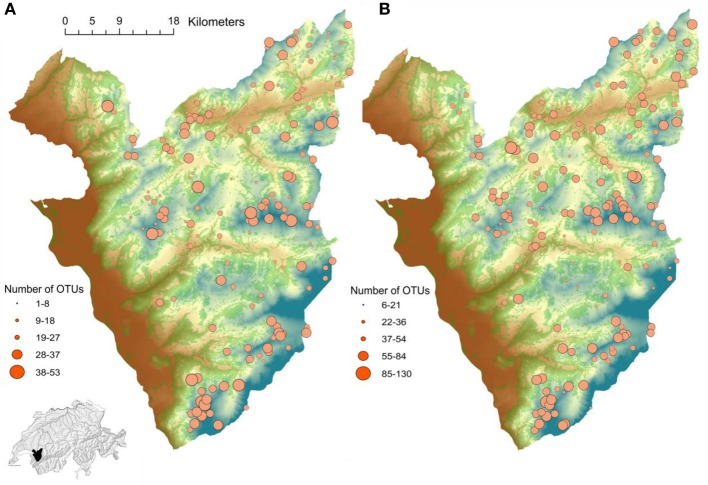
**Study area in the Western Swiss Alps (700 km^2^)**. The gradient from brown to blue represents elevation (400–3210 m). The green surface represents forested areas. The dot size corresponds to the number of OTUs per plot of **(A)** Glomeromycetes and **(B)** Agaricomycetes in the inventoried plots.

A dataset of 213 communities of plant and soil fungi was collected in the Western Swiss Alps (Figure 1), spanning an elevation gradient from 400 to 3210 m above sea level, to improve our understanding of spatial variation in fungal communities composition (see Pagni et al., 2013). Exhaustive inventories of all vascular plant species were conducted at each sampled location within 4 m^2^ plots (Dubuis et al., 2013). Five soil samples were collected from each vegetation plot (the four corners and the center of each plot) for DNA extraction and soil chemical analyses (See Appendix). The composition of soil fungal communities was determined by pyro-sequencing of fungal internal transcribed spacer 1 (ITS1) amplicons, generated with the universal fungal primer pairs ITS1F—ITS2 (Gardes and Bruns, 1993) from each sample of soil DNA. Once parsed and clustered into Operational Taxonomic Units (OTUs) using the DBC454 hierarchical clustering algorithm (described in details in Pagni et al., 2013), the number of OTUs per plot was defined.

Across the sampled plots, OTUs from 14 taxonomic classes were found, including two classes potentially interacting with plants, Glomeromycetes and Agaricomycetes. In order to have a more complete overview of AMF assemblage structure in soils, a partial region of the small subunit of ribosomal DNA gene (SSU), generated with the Glomeromycetes (AMF) specific primers pairs NS31- AM1 (Helgason et al., 1999), was also sequenced. The composition of these communities will be presented elsewhere. We retain here only the information concerning the number of OTUs of Agaricomycete and Glomeromycetes. That of Agaricomycetes was estimated by the number of ITS1 OTUs per plot as summed across subplot assigned to this fungal class, whereas that of Glomeromycetes was estimated by the number of SSU OTUs per plot.

Using species distribution models (SDMs), we tested whether including a predictor representing the number of fungal OTUs potentially interacting with plants (i.e., mutualists or pathogens) allowed fitting statistical models better describing the distributions of plant species. SDMs typically relate presence and absence of a given species to a set of environmental parameters (Guisan and Zimmermann, 2000). We ran generalized linear models (GLM) with binomial distribution and a logistic link function on 124 plant species (those with more than 10 occurrences across the sampled sites) by taking into account seven abiotic factors [degree-days, moisture, solar radiations, soil pH, soil phosphate, slope, and topography as done in Dubuis et al. (2012, 2013)]. We ran GLMs including the number of OTUs of Glomeromycetes and Agaricomycetes obtained per plot. We validated the models performance using 10-fold cross-validation measured by the area under the Receiver Operating Characteristic (ROC) curve (area under the curve, AUC). We compared model fit estimated with the adjusted explained deviance (*R*^2^). We computed the *R*^2^ of the global fit, as well as, the AUC with and without the fungi biotic predictor.

### Results

**Figure 2:**
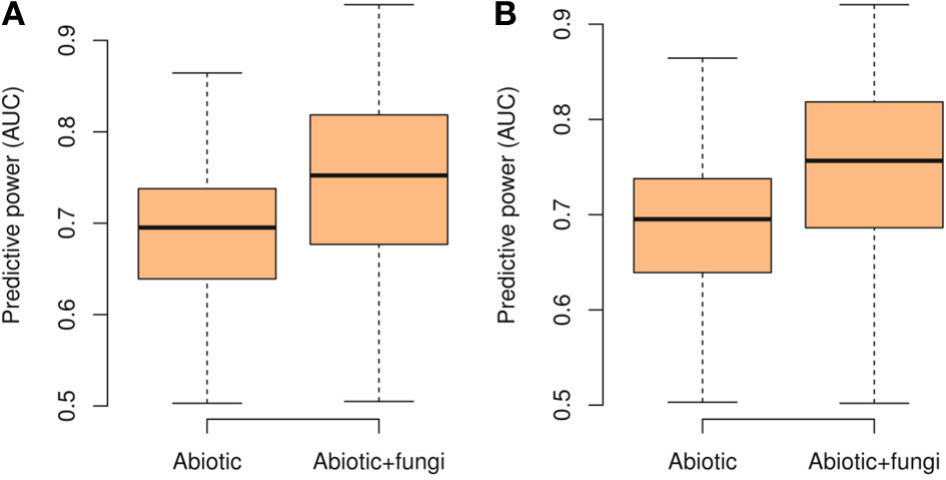
**Boxplot of the predictive power of the species distribution models measured with the area under the curve (AUC) when considering only abiotic predictors or additionally including also the number of OTUs of potentially mutualistic fungi**. Shown are the results for Glomeromycetes **(A)**and Agaricomycetes **(B)**.

**Figure 3:**
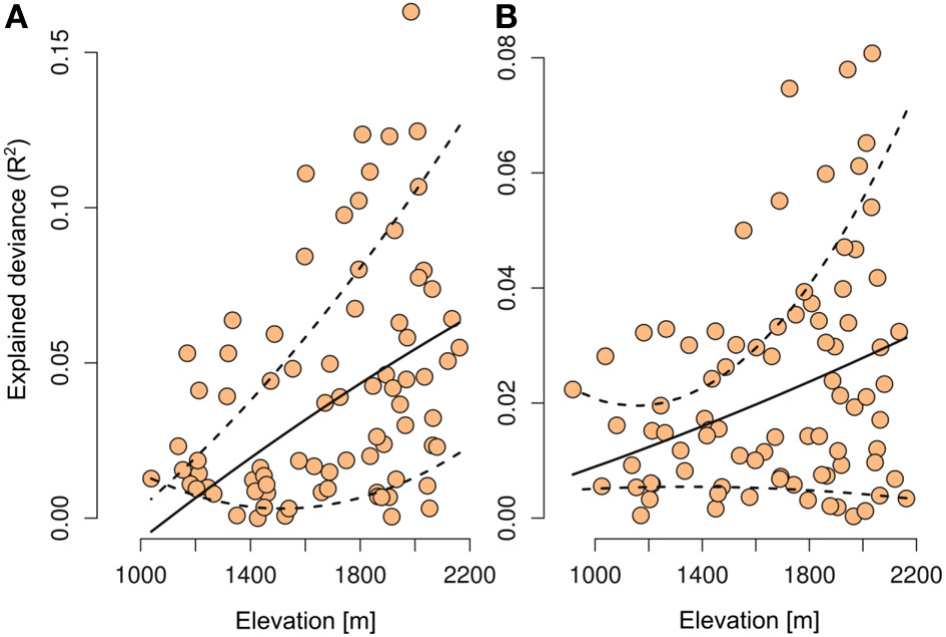
**Relationship between the average elevation where a plant species is found and the explained deviance of the models when only the number of OTUs is included in the species distribution models**. Shown are the results with Glomeromycetes **(A)** and Agaricomycetes **(B)** when considered alone as predictor in the species distribution models. The dashed lines represent the 10th and 90th percentile confidence interval from quantile regression.

When the number of OTUs was included in the plant distribution models, we found a significant increase of explained deviance (*R*^2^, paired Wilcoxon test, Glomeromycetes *V* = 1306, *p* < 0.0001, Agaricomycetes *V* = 1326, *p* < 0.0001) and predictive power (AUC, paired Wilcoxon test, Glomeromycetes *V* = 6713, *p* < 0.0001, Agaricomycetes *V* = 7585, *p* < 0.0001, Figure 2). Even after including the abiotic drivers most likely to drive plant species distributions in the models (i.e., topography, climate, and soil), information on potential mutualistic fungi in the soil consistently increased the predictive accuracy of plant SDMs (Figure 2). Thus, the number of OTUs of Agaricomycetes and Glomeromycetes fungi in the soil provides relevant additional information to explain plant species distributions. As Agaricomycetes also include other functional groups than EMF, such as decayers and pathogens, these latter functions may also play a role in the observed signal in our analyses. For each plant species, we also computed the elevation average of the site where the species was found. In parallel, we computed the explained deviance (*R*^2^) of the GLMs, when the number of OTUs was included alone in the models of plant species distributions. We found that those plant species with the strongest relationship (*R*^2^) with the number of fungal OTUs occurred at higher elevations (*t*-test, Glomeromycetes: *t* = 4.01, *p* = 0.0001, Agaricomycetes: *t* = 2.97, *p* = 0.03; Figure 3).

High elevation soil is especially variables in the number of OTUs (Figure 1) and fungal variation among communities may impact plant composition. In particular, it was shown that even if mycorrhizal associations are still reasonably frequent in alpine environments, the level of colonization differs greatly between habitats (Read and Haselwandter, 1981). The differential composition of mutualist fungi across alpine habitats may thus play a role in modulating the composition of plant communities. The benefits that plants derive from association with mycorrhizal fungi might be especially important for high mountain plant species growing in harsh environments. This may be because high elevation environments are characterized by poorer soils and harsher environmental conditions (Körner, 2003), rendering the establishment and growth of plant species more hazardous and making symbiosis with microorganisms that provides nutrients missing in the soil more beneficial. Supporting our results, Wagg et al. (2011) examined seedling growth from two *Pinus* species at two elevations. Low elevation soil was the most fertile with a complex soil microbial community, but the latter had an overall negative effect on seedling growth. In contrast, high elevation soil was the least fertile but had a microbial community that enhanced seedling growth. This suggests that in harsh environment with lower productivity such as high elevations, dependence of plants on soil microbes may be greater (Johnson et al., 1997; Thrall et al., 2007). However, as fungal diversity may vary across high elevation habitats, it may impact the establishment of plant communities.

Several studies indicated a decrease in mycorrhizal fungal diversity associated with plant roots with increasing elevation (Bahram et al., 2012; Gai et al., 2012; Lugo et al., 2012). While those studies have documented changes in diversity of mycorrhizal fungi with elevation, no experimental study has yet investigated the importance of mycorrhizal fungi for plant establishment along an elevation gradient. In a calcicolous alpine area similar to the study region, Blaschke (1991) showed that mycorrhizal fungi were commonly recorded on the roots of alpine flora, even if intensities and patterns of colonization by mycorrhizal fungi varied among species. Therefore, even if the overall diversity of mycorrhizal fungi decreases with elevation, it does not necessarily suggest a lower importance of mycorrhizal fungi for plant establishment, but may imply more persistent relationships.

Even if mycorrhizal associations are still reasonably frequent in alpine environments, the level of colonization differs greatly between plant species and habitats (Read and Haselwandter, 1981). This explains why some species distributions showed a relationship with the number of fungal OTUs, while others did not (Figure 3). In addition, Blaschke (1991) suggested that mycorrhizal colonization was greatest where spatial clusters of suitable host plants (i.e., host plant guilds) were formed, suggesting facilitation of colonization due to the proximity of roots with established mycorrhizal associations. Mycorrhizal colonization is generally positively correlated with host plant density, and a decrease in vegetation cover toward higher elevation may also affect mycorrhizal colonization of plant roots (Hartnett et al., 1993; Genney et al., 2001; Ruotsalainen and Väre, 2004). Hence, fungi may influence the establishment of alpine plants, but the structure of plant communities may also enhance the colonization of plant root systems by fungi through feedback mechanisms.

## Limitations of the correlative approach when studying plant-fungal interactions

A first limitation arises from the fact that, because the approach proposed here is correlative, we cannot exclude the possibility that the inclusion of significant descriptors of AMF and ECM fungi in the models indirectly reflected a missing abiotic component (Wisz et al., 2013). Many abiotic factors influence plant species establishment and growth in a community. This means that identifying and measuring the entire set of abiotic conditions driving plant community assembly is particularly challenging. It is relatively easy to miss some important abiotic predictors of plant distribution (Austin and Van Niel, 2011). In particular, we measured soil moisture as the ratio between precipitation and potential evapo-transpiration (Zimmermann and Kienast, 1999), since direct measures of soil moisture are still difficult to conduct in a standardized way across large areas (Austin and Van Niel, 2011). Yet, soil moisture is considered a strong driver of plant species distributions, even in cold environments (Le Roux et al., 2013). Since fungi are also expected to strongly depend on soil moisture, the diversity of mycorrhizal fungi may indirectly inform on the moisture conditions at the site, but this would remain to be tested with better soil moisture measurements in the field.

Because mycorrhizal colonization percentage is generally positively correlated to host plant density (Genney et al., 2001), mycorrhizal fungal diversity may also inform on the competitive pressure between plant species for establishment and persistence. Inter-specific plant competition may be an important factor shaping species distributions even in a cold environment (Pellissier et al., 2010a). Fungal assemblage in soil may correlate with plant density (Gilbert et al., 2002). Since in condition of high density, competition among plant species is expected to increase and lead to exclusion from communities, the effect of fungi on plants may be confounded with plant competitive effects. To dissect a potential confounding effect of plant density, we tested the relationship between the sum of plant cover and the number of OTUs. We found no relationship between the number of fungal OTUs and the sum of plant cover in a given plot (Coefficient of determination, Glomeromycetes *R*^2^ = 0.06, Agaricomycetes *R*^2^ = 0.04). The relationship between fungi and plant distribution is thus independent from a potentially confounding effect of plant density. However, we did not consider plant belowground competition as such a measure is difficult to quantify in the field. As a consequence, this confounding effect cannot be fully excluded.

Another limitation of the analysis results from the low resolution of the OTUs assignment. The OTUs identified from the ITS reads were assigned at a coarse taxonomic level, which does not let us know to which functional group they belong. Thus, while we expect a consistent mycorrhizal function for Glomeromycetes, Agaricomycetes regroup species from distinct functional groups, including decayers, plant pathogens and mutualists. As a consequence, the richness of Agaricomycetes OTUs in soils cannot be linked directly to a function, and must be interpreted with caution. Therefore, even if we found a biological signal in our analyses, we cannot conclude on the functional link that ties fungi diversity to plant distribution. In this regard, correlative approaches provide interesting testable hypotheses, but only experimental designs can ultimately unravel the causality behind plant-fungi relationships.

## Conclusion and perspectives

Our literature review highlighted our lack of knowledge about the spatial variation in interactions between plants and mycorrhizal fungi along varying ecological conditions. Most studies so far have limited their scope to a few communities under standard environmental conditions and highlighted the role of mycorrhizal fungi in affecting plant community composition. However, some evidence suggests that the role of mycorrhizal fungi may extend beyond single communities and may shift in importance along wide environmental gradients. Our results support the role of soil fungal assemblage in driving plant species distributions at the landscape scale, especially at high elevations, but also call for a more controlled approach to the problem. Manipulative field experiments will be required that consider the manipulation of both plants and mycorrhizal fungi to learn more concerning their interactions along environmental gradients. To our knowledge, no study has yet designed spatially explicit experiments that could formally demonstrate the role of fungi in driving the spatial distribution of plant species. This would require the manipulation of fungal communities in the field, either with fungal-addition treatments (Mendes et al., 2010), fungal-removal treatments using fungicides (Helgason et al., 2007), or a combination of both. However, field manipulation of micro-organisms is challenging and complementary correlative approaches such as used here will likely remain valuable to garner information on plant-fungi interactions at wider spatial scales.

### Conflict of interest statement

The authors declare that the research was conducted in the absence of any commercial or financial relationships that could be construed as a potential conflict of interest.

## Appendix

The sampling was conducted in the Western Swiss Alps (Figure 1). The elevation gradient of the region spans the altitude from 400 to 3210 m. Following a balanced random-stratified sampling design, based on elevation, slope and aspect we sampled 213 plots across a variety of non-forested vegetation. Exhaustive inventories of all vascular plant species were conducted at each sampled location within 4 m^2^ plots. Five soil samples were taken per vegetation plot (from the four corners and one from the middle of each plot) for DNA extraction and soil chemical analysis. For the soil chemical analysis, the five samples were mixed to equalize eventual intra-plot variation. After drying the samples, we measured the pH, soil texture, total nitrogen (N), total phosphorous (P), and organic carbon (C) (for details on the specific methods see Dubuis et al., 2013).

We computed climatic predictors over the area from data on the monthly means of the average temperature (°C) and sum of precipitation (mm) recorded for the period of 1961–2010 by the Swiss network of meteorological stations interpolated using a digital elevation model at 25 m resolution. We computed degree-days as the sum of days multiplied by the temperature above 0°C, and moisture index as the difference between precipitation and potential evapotranspiration for the growing season of June, July, and August (Zimmermann and Kienast, 1999). Topographic variables were obtained from a digital elevation model.

### PCR conditions and pyrosequencing

Soil samples were independently homogenized and sieved with a 2 mm sieve. 3 g of soil of each sample was air dried at 30°C, and genomic DNA was extracted from 250 mg of dry soil following the recommendations of the PowerSoil®-htp 96 Well Soil DNA Isolation Kit distributor (MoBio Laboratory, Carlsbad, USA). The universal fungal primers ITS1F and ITS2 have been chose to amplify the total fungal community present within the samples, also the Glomeroycota specific primers NS31 et AM1 have been used to amplify a partial region of the small subunit of ribosomal DNA (SSU) gene. ITS1F and NS31 were linked to the Genome Sequencer FLX Titanium pyrosequencing primer A, also ITS2 and AM1 were linked to the pyrosequencing primer B. A barcode key of 10 bp in length was inserted between the A primer and ITS1F or NS31 primer sequence. Thus, the composite forward primer was: 5′-CGTATCGCCTCCCTCGCGCCATCAG–(X)10-*CTTGGTCATTTAGAGGAAGTAA*-3′/5′-CGTATCGCCTCCCTCGCGCCATCAG–(X)10- *TTGGAGGGCAAGTCTGGTGCC*-3′, respectively. The reverse primer was 5′-CTATGCGCCTTGCCAGCCCGCTCAG-*GCTGCGTTCTTCATCGATGC*-3′/5′-CTATGCGCCTTGCCAGCCCGCTCAG-*GTTTCCCGTAA*GGCGCCGAA -3′, where A and B primers are underlined, the barcode key is indicated by (X)10 and the fungal specific primers are shown in italics. A set of 162 different barcode keys of 10 bp each, designed according to Roche indications, were used. The main criterion of these barcode keys is that at least 3 nucleotides out of 10 have to differ between all the barcodes used. The ITS1 PCR reactions were performed as described in Buée et al. (2009), also the SSU gene PCR reactions were performed as described in Opik et al. (2009). The amount of DNA in the PCR products was measured using NanoDrop 1000 (Thermo Scientific, Wilmington, DE, USA). Amplicons with different barcode keys were pooled in equimolar proportions. Thus, 6 amplicon libraries of 167 samples each have been generated for each marker (ITS1 or SSU). Then, the amplicon libraries were separated by electrophoresis through a 1.5% agarose gel in 0.5 × TBE, and the PCR amplicons were purified from the gel using the Qiagen QIAquick Gel Extraction kit (Qiagen, Switzerland). The amount of DNA in the purified amplicon libraries was measured using NanoDrop 1000 (Thermo Scientific, Wilmington, DE, USA). Pyrosequencing of the six ITS1 amplicon libraries (from the ITS1F primer) and 6 SSU amplicon libraries (from the NS31 primer) on the Genome Sequencer FLX Titanium 454 System (454 Life Sciences/Roche Applied Biosystems, Nutley, NJ, USA) was performed at Microsynth (Balgach, Switzerland).

From the total reads obtained, 70% of sequences corresponded to the full length. All details of the bioinformatics analysis and benchmarking is given in Pagni et al. (2013). As each cluster generated by DBC454 contain reads identified in the present project and reads described in Genbank, the procedure allows the assignment of the reads contained in each cluster to a taxonomic group. Also, the number of OTUs for each taxonomic group per plot including Glomeromycetes (obtained via the SSU marker) and Agaricomycetes (obtained via the ITS1 marker) can be extracted for further statistical analysis.
